# Mesenchymal stem cells derived from breast cancer tissue promote the proliferation and migration of the MCF-7 cell line *in vitro*

**DOI:** 10.3892/ol.2013.1619

**Published:** 2013-10-11

**Authors:** CHUNFU ZHANG, WEI ZHAI, YAN XIE, QIAOLIN CHEN, WEI ZHU, XIAOCHUN SUN

**Affiliations:** 1 The Second People’s Hospital of Kunshan, Kunshan, Jiangsu 215300, P.R. China; 2 School of Medical Science and Laboratory Medicine, Jiangsu University, Zhenjiang, Jiangsu 212013, P.R. China

**Keywords:** breast cancer mesenchymal stem cells, proliferation, migration

## Abstract

Mesenchymal stem cells (MSCs) are critical in promoting cancer progression, including tumor growth and metastasis. MSCs, as a subpopulation of cells found in the tumor microenvironment, have been isolated from several tumor tissues, but have not been isolated from breast cancer tissue to date. Therefore, the purpose of this study was to isolate MSCs from primary human breast cancer tissue, and to study the effect of breast cancer MSCs (BC-MSCs) on the proliferation and migration of the MCF-7 cell line *in vitro*. MSCs were isolated and identified from primary breast cancer tissue obtained from 9 patients. The MCF-7 cell line was treated with 10 and 20% breast cancer-associated MSC (BC-MSC)-conditioned medium (CM) for 10–48 h, and changes in proliferation and migration were observed. Furthermore, we investigated the migration of 10 and 20% CM concentrations on MCF-7 through a scratch wound assay and a transwell migration assay. We successfully isolated and identified MSCs from primary breast cancer tissues. BC-MSCs showed characteristics similar to those of bone marrow MSCs, and possessed the capability of multipotential differentiation into osteoblasts and adipocytes. The results of the 3-(4,5-dimethylthiazol-2-yl)-2,5-diphenyltetrazolium bromide (MTT) assay showed that 10 and 20% CM concentrations increased the proliferation of MCF-7 cells to different levels. The results also revealed a greater increase in different levels compared with the control group. In conclusion, MSCs were confirmed to exist in human breast cancer tissues, and BC-MSCs may promote the proliferation and migration of breast cancer cells.

## Introduction

The high incidence of breast cancer in modern society is a serious threat to women’s health. Approximately 30% of early stage breast cancer patients eventually develop recurrence and metastasis (1). Therefore, targeted breast cancer cell research and treatment is of great importance. Mesenchymal stem cells (MSCs) are non-hematopoietic multipotent cells that may be isolated and expanded from a number of different sources, including bone marrow and adipose tissue (2,3). They are defined by a series of specific cell surface antigens (4) and an inborn ability to differentiate along multiple lineages, including into osteoblasts, chondrocytes and adipocytes (5–8). Although MSCs have a primary role in tissue regeneration (9,10), they also possess the ability to migrate to the site of numerous tumor types *in vivo*(11,12). A recent study strengthened the link between MSCs and carcinoma (13). The interaction between cancer cells and the tumor microenvironment is increasingly being regarded as an important regulator of malignant progression. It is well-known that tumor cells secrete chemokines, cytokines and growth factors that are able to recruit and activate a number of MSCs. In turn, MSCs, as a part of the tumor microenvironment secrete cytokines and chemokines to affect the growth and metastasis of tumor cells (14,15). MSCs are able to selectively target tumor sites. Indeed, actively growing tumors recruit MSCs in their environment where they promote tumor growth and metastasis to distant organs (16,17). There is ample evidence that MSCs may be isolated from tumors such as lipoma (18), bone sarcoma (19) and uterine cervix cancer (20). Cao *et al*(21) identified that MSC-like cells in human gastric cancer tissues were similar to bone marrow (BM)-MSCs in their morphology, surface antigens, specific gene expression and differentiation potential. Lis *et al*(22) successfully isolated ovarian cancer-associated MSCs and found that they could protect ovarian cancer cells from hyperthermia by secreting CXCL12. Another study showed that BM-MSCs could facilitate breast cancer cell metastasis through the secretion of CC chemokine ligand 5 (CCL5) (14). These results indicate that the interaction between MSCs and cancer cells may represent an important therapeutic target for the prevention of cancer progression. Overall, these studies show that MSCs in tumor tissues may be important in modulating cancer cell proliferation and metastasis. Relatively little is known regarding whether MSCs are located in primary breast cancer tissue, and how they participate in breast cancer proliferation and migration.

In the present study, we successfully isolated MSCs from human breast cancer tissue and investigated the effect of breast cancer MSCs (BC-MSCs) on the MCF-7 breast cancer cell line. The results confirm the existence of MSCs in human breast cancer tissue, and indicate that BC-MSCs may promote the proliferation and migration of the MCF-7 breast cancer cell line *in vitro*.

## Materials and methods

### Cell culture

Cells were cultured as described previously (23,24). Cells were maintained in Dulbecco’s modified Eagle’s medium with low glucose (L-DMEM) supplemented with 10% fetal bovine serum (FBS), 100 U/ml penicillin and 100 U/ml streptomycin, under mycoplasma-free conditions at 37°C in a humidified atmosphere of 5% CO_2_ and 95% air.

### Isolation and culture of BC-MSCs

Cancer tissue was obtained from patients (n=9) with breast carcinoma who had undergone mastectomies at The Second People’s Hospital of Kunshan (Kunshan, China). All patients agreed voluntarily to participate in the study subject to the terms agreed by the Ethics Committee of Jiangsu University. Fresh tissue specimens were collected and washed with phosphate-buffered saline (PBS). The tissue specimens were cut into 1 mm^3^-sized pieces and floated in L-DMEM containing 10% FBS, 100 U/ml penicillin and 100 U/ml streptomycin. The specimens were subsequently incubated at 37°C in humid air with 5% CO_2_. The medium was replaced every three days after the initial plating. When adherent fibroblast-like cells appeared after 10 days of culture, the cells were trypsinized and passaged (without dilution) into a new flask for further expansion. The cells in passage 3 were used for the evaluation of the experimental results.

### Flow cytometry

Flow cytometric analyses were performed at passages 3 and 4. BC-MSCs (1.0×10^6^ cells) were trypsinized, washed twice in PBS and stained with monoclonal antibodies against CD13, CD29 and CD71 (PE-conjugated); and CD4, CD10, CD14, CD34, CD38, CD44, CD105, HLA-DR and HLA-I (FITC-conjugated) (Becton-Dickinson, San Jose, CA, USA) for 30 min on ice. Labeled cells were analyzed using a FACSCalibur flow cytometer (Becton*-*Dickinson). PE-IgG1 and FITC-IgG1 were used in the control group.

### Osteogenic and adipogenic differentiation in vitro

BC-MSCs were seeded at 5,000 cells/cm^2^ in 35-mm plates and cultured in L-DMEM with 10% FBS, and either osteogenic [0.1 μM dexamethasone, 10 mM b-glycerophosphate, 50 mg/l ascorbic acid and 4 μg/ml basic fibroblast growth factor (bFGF) (Sigma-Aldrich, St. Louis, MO, USA)] or adipogenic (10^−6^ M dexamethasone, 0.5 μM isobutylmethylxanthine, 5 ng/ml linsulin, 60 μM indomethacin and 10^−4^ M hydrocortisone) supplements (Cyagen Biosciences, Sunnyvale, CA, USA). The medium was changed three times a week and the cells were induced for two weeks. At the end of induction, the cells were subjected to alkaline phosphatase staining and Oil Red O staining followed by hematoxylin counterstaining.

### Reverse transcription polymerase chain reaction (RT-PCR)

RNA was extracted from ~1.0×10^6^ MSCs, differentiating cells or differentiated cells using TRIzol reagent (Invitrogen, Carlsbad, CA, USA). RNA (1 μg) was processed for cDNA synthesis with Superscript II reverse transcriptase, using Oligo(dT) primer (Toyobo, Osaka, Japan) according to the manufacturer’s instructions. PCR was performed using 1 μg of cDNA sample with 0.3 units of Taq polymerase (CinnaGen, Tehran, Iran), 200 μM dNTPs, 10 pM of each primer, reaction buffer and MgCl_2_ (Takara, Shiga, Japan) in a 25 μl PCR tube. The PCR amplification was performed for 35 cycles using an ABI 2720 thermal cycler (Applied Biosystems, Foster City, CA, USA). The cycling conditions were as follows: 94°C for 30 sec, 60°C (primer) for 30 sec and 72°C for 30 sec, with a final extension at 72°C for 10 min, respectively. PCR products were separated on a 1.5% agarose gel, stained with ethidium bromide and visualized under UV light. For PCR, the forward and reverse primers were as follows: BMP-3, 5′-GACCCTCCAATCCAACCA-3′ (forward) and 5′-ACGCTTTCAGGCTCACAA-3′ (reverse); peroxisome proliferator-activated receptor γ-2 (PPARγ-2), 5′-GCCCAGGTTTGCTGAATG-3′ (forward) and 5′-TGAAG ACTCATGTCTCTC-3′ (reverse); glyceraldehyde 3-phosphate dehydrogenase, 5′-GGATTTGGTCGTATTGGG-3′ (forward) and 5′-GGAAGATGGTGATGGGATT-3′ (reverse).

### Generation of conditioned media

Conditioned media was generated based on previously published methods (25,26). BC-MSCs were plated to 70% confluency in 35-mm plates with 10% FBS L-DMEM and allowed to adhere overnight at 37°C and 5% CO_2_. The following day, the media was removed and the cells were washed twice with PBS. Cells were then re-incubated with non-serum culture media. After 24 h, the media was collected, spun down to remove cell debris (698 × g for 5 min) and passed through a 0.45 μm filter (Sigma-Aldrich). CM aliquots were frozen at −20°C until required (not exceeding two weeks). To prepare different concentrations of BC-MSC-CM (10 and 20%), the near 100% BC-MSC-CM was diluted accordingly in freshly prepared L-DMEM with 10% FBS.

### 3-(4,5-dimethylthiazol-2-yl)-2,5-diphenyltetrazolium bromide (MTT) assay

Cells were plated at a density of 2.5×10^3^ cells/well in a 96-well plate in 200 μl 10% FBS L-DMEM, and allowed to attach overnight. Cells were then treated with 10 and 20% BC-MSC-CM for 48 h. MTT (20 μl) was added to each well for the last 4 h. When the reaction was terminated, all the solution was discarded and 150 μl of dimethyl sulfoxide was added to each well. The 96-well plate was shaken to ensure complete solubilization of the purple formazan crystals. Absorbance at 490 nm was measured by an enzyme-linked immunosorbent assay reader.

### Scratch wound assay

Cells were grown to confluence and then scratched with a cell scraper (Nunc, Inc., Naperville, IL, USA). The resulting debris was removed by gentle washing with medium. The cells were subsequently placed in an incubator. Cells were maintained for up to 24 h with or without CM. The images of the closing wound were acquired by inverted microscopy and analyzed using ImageJ software (National Institute of Health, Bethesda, MD, USA).

### Transwell migration assay

Migration assays were performed based on the study by Karnoub *et al*(14) and the manufacturer’s instructions (Corning Inc., Corning, NY, USA). There was conditioned medium (0, 10 and 20%) in the bottom of the transwell. A volume of 5×10^4^ MCF-7 cells was plated in 100 μl of serum-free L-DMEM in the top of the chamber and incubated for 10 h at 37°C. The cells on the top side of the filter were removed by scrubbing twice with a cotton-tipped swab. Migrating cells were fixed in formaldehyde and stained with crystal violet. Four lower power fields (magnification, ×100) were randomly selected in each chamber to observe the cells and Cell Counter software (Borland Software Corporation, Scotts Valley, CA, USA) was used to count the stained migrated cells. Each experimental group was repeated three times.

### Statistical analysis

Studies involving more than two groups were analyzed by one-way ANOVA with Newman-Keuls multiple comparison test, using the GraphPad Prism V.5 software program. The results were expressed as the means ± SD from three different replicates for individual assays. P<0.05 was considered to indicate a statistically significant difference.

## Results

### Morphological characterization and identification of BC-MSCs

**Figure 1:**
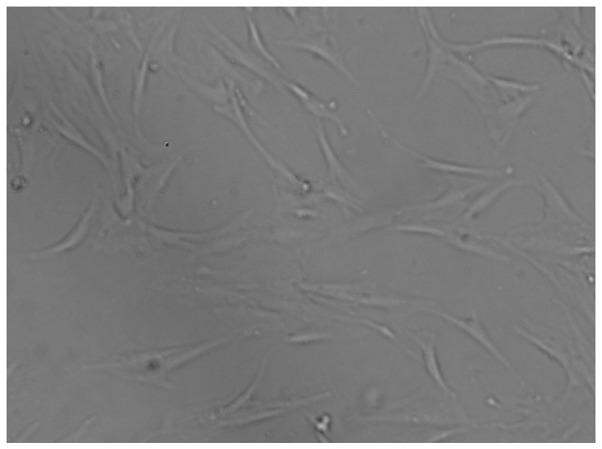
BC-MSCs were long, spindle-shaped and fibroblastic in appearance after 10 days of primary culture. Magnification, ×200. BC-MSCs, breast cancer mesenchymal stem cells.

**Figure 2:**
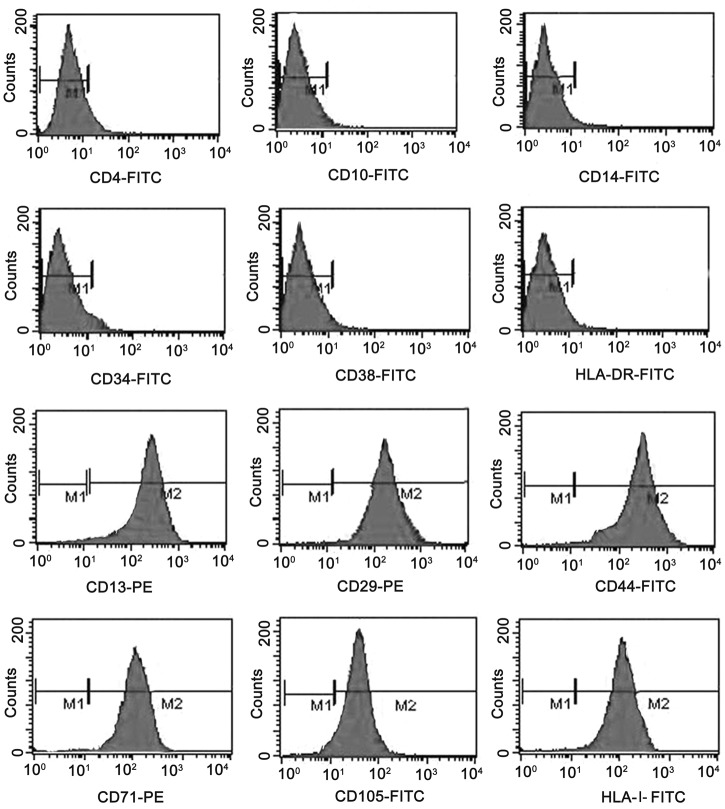
Surface antigens of BC-MSCs. BC-MSCs were positive for CD13, CD29, CD44, CD71, CD105 and HLA-I, but negative for CD4, CD10, CD14, CD34, CD38 and HLA-DR. BC-MSCs, breast cancer mesenchymal stem cells.

After the initial 3–5 days of primary culture, a small population of single cells with a spindle shape were observed, which had adhered to the plastic surfaces. On days 7–10 after the initial plating, the cells were displayed as long spindle-shaped or polygonal fibroblastic cells (Fig. 1). Flow cytometry analysis demonstrated that the BC-MSCs possessed uniform surface markers. The BC-MSCs expressed the same surface antigens as human BM-MSCs, and they were positive for CD13, CD29, CD44, CD105 and HLA-I, but negative for CD4, CD10, CD14, CD31, CD34, CD38 and HLA-DR. MSCs isolated from the bone marrow of healthy adult donors were used as a positive control (Fig. 2).

### Multilineage differentiation potential of BC-MSCs

**Figure 3:**
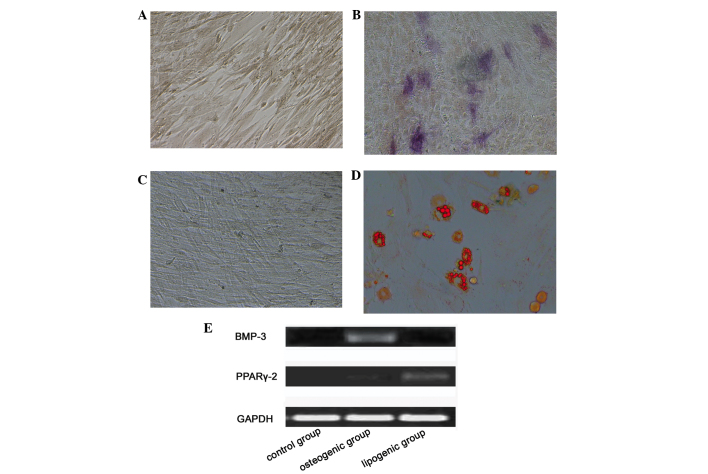
Differentiation potential of BC-MSCs. (A) Osteogenic control group. (B) Results of ALP detection in cell cultures grown for 2 weeks in osteogenic medium. Part of the MSCs became ALP positive. (C) Adipogenic control group. (D) Results of Oil Red O staining detection in cell cultures grown for 2 weeks in adipogenic medium. Part of the cells contained numerous Oil Red O-positive lipid droplets. (A–D) Magnification, ×200. (E) Cells expressed BMP-3 and peroxisome proliferator-activated receptor γ-2 (PPARγ-2) gene after being induced, respectively. BC-MSCs, breast cancer mesenchymal stem cells; ALP, alkaline phosphatase.

*In vitro* multilineage differentiation potential is the functional standard for verifying the identity of MSCs. Differentiation of BC-MSCs was apparent after two weeks of induction in the special medium. At the end of the second week, both BC-MSCs were capable of differentiation into either osteocytes or adipocytes, shown by the positive staining of alkaline phosphatase (Fig. 3A and B) and Oil Red O (Fig. 3C and D). With osteogenic supplementation, the differentiation was apparent after incubation. RT-PCR results showed that these cells highly expressed BMP-3 (Fig. 3E). Similarly, after adipogenic induction, the cells expressed PPARγ-2 (Fig. 3E). Non-treated control cultures did not notably express BMP-3 and PPARγ-2.

### BC-MSC-CM enhances the proliferation of MCF-7 breast cancer cells in vitro

**Figure 4:**
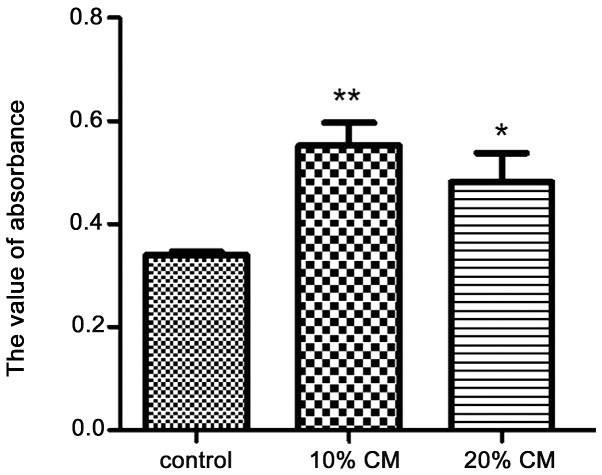
Analysis of the proliferation activity of MCF-7 cells. CM, conditioned medium.

For the MTT assay, MCF-7 cancer cell lines cultured in BC-MSC-CM (10 and 20%) showed an increase in cell proliferation. Compared with the control group, MCF-7 cells were increased by 164 and 137%, respectively (Fig. 4). The increase in cell proliferation observed for MCF-7 was statistically significant compared with that of the control group.

### BC-MSCs promote the migration of MCF-7 breast cancer cells in vitro

**Figure 5:**
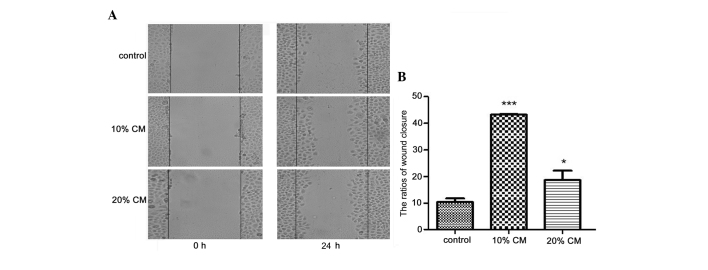
Cell scratch test for cell migration. (A) Exemplary microphotographs of wound closure in control, 10% CM and 20% CM groups. (B) Ratios of wound closure in the different groups. There was a significant difference between the 10% CM group and the 20% CM group in terms of the speed of wound healing when compared with the control group (P<0.05). CM, conditioned medium.

**Figure 6:**
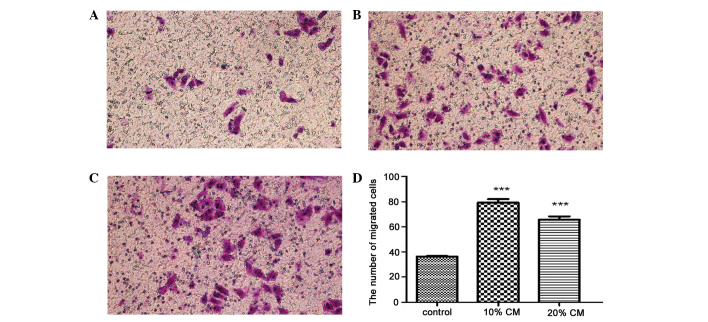
Transwell migration assay. (A) Control, (B) 10% CM and (C) 20% CM groups. (D) The number of migrated cells in the different groups. There was a significant difference between the 10% CM group and 20% CM group in terms of the number of migrated cells when compared with the control group (P<0.05). CM, conditioned medium.

Scratch wounds were inflicted on cells pre-treated with or without BC-MSC-CM for 24 h. The surface area of the wounds generated did not differ between the groups at 0 h, while differences were observed between the groups after 24 h of treatment (Fig. 5A). The wound closure ratios were 10.2±1.3, 44.0±0.3 and 18.5±3.1% for MCF-7 cells in the control group, 10% BC-MSC-CM group and 20% BC-MSC-CM group, respectively, with the data showing statistical significance (Fig. 5B). In this study, we set out to determine whether BC-MSCs affect the migration potential of the normally non-metastatic MCF-7 cell line. In the first 24 h of culture, the cell migration assay showed cell migration in the MCF-7 cancer cell line stimulated with 10 and 20% BC-MSC-CM after overnight starvation in serum-free medium. After 8 h of culture, the migration of MCF-7 cells was enhanced by BC-MSC-CM (10 and 20%) compared with that of the control group (Fig. 6A–C). A greater number of viable cells migrated into the lower chamber of the transwell following treatment with BC-MSC-CM (10 and 20%) compared with that of the controls, as indicated by the viability assay. The mean numbers of migrated cells per lower power fields were 38.3±0.6, 81.7±2.1 and 62.3±1.5, respectively. There were significant differences between the control group and the BC-MSC-CM groups (Fig. 6D). The results showed that treatment with BC-MSC-CM greatly increased the ability of the MCF-7 cells to migrate to the lower side of the well.

## Discussion

In contrast to research on cancer-associated MSCs obtained from the bone marrow of hematological malignancies (27), MSCs derived from solid tumors have not been studied in detail and their role in cancer progression remains poorly defined. A detailed characterization of their role in human cancer progression would help to clarify the potential targets for cancer therapy.

Previous studies that have detected the effects of human MSCs (hMSCs) on primary carcinoma cells have resulted in conflicting findings (23,28–30), and no apparent effects of MSCs on cancer progression have been reported. It is possible that MSCs derived from cancer tissue may be affected by the tumor microenvironment and, in turn, affect the tumor cells (31). Although it has been reported that MSCs may support tumor growth (15) and promote cancer metastasis (14), further study on the detailed role of MSCs in tumor progression and its mechanisms is still required in various models.

In this study, BC-MSCs from human breast cancer tissues showed a homogenous immunophenotype and a multi-lineage differentiation potential (osteoblast and adipocyte) under appropriate conditions. BC-MSCs grew rapidly and showed fibroblastic morphology. We demonstrated that they were homogeneously positive for the mesenchymal cell markers CD13, CD29, CD44, CD71, CD105 and HLA-I, but negative for CD4, CD10, CD14, CD34, CD38 and HLA-DR.

To investigate the differentiation potential of BC-MSCs, we used the third passage from BC-MSCs for culturing in the conditions that favored the osteogenic and adipogenic differentiation of MSCs. The results showed that BC-MSCs were alkaline phosphatase- and Oil Red O-positive after being induced. Furthermore, RT-PCR results showed that these cells highly expressed osteogenic and adipogenic marker genes, such as BMP-3 and PPARγ-2. Therefore, our experiments revealed that BC-MSCs may be induced to differentiate into bone and fat *in vitro*. The results demonstrated the successful isolation and identification of canonical MSCs in primary breast cancer tissue.

Furthermore, we observed the effects of BC-MSCs on the proliferation and migration of the human breast cancer cell line, MCF-7, *in vitro*. We selected the CM from the BC-MSCs to culture MCF-7 rather than create a co-culture. Cell co-culture studies are occasionally unreliable due to possible artificial growth advantages of one cell type over the other induced by the culture environment rather than by a genuine anticancer effect (32). To rule out this possibility, we focused on the role of the BC-MSC-CM rather than of the direct cells in the present study. The effect of BC-MSC-CM on MCF-7 *in vitro* was examined using MTT cell proliferation. The results of MTT cell proliferation showed that BC-MSC-CM significantly stimulated cancer cell proliferation. Therefore, this indicates that BC-MSC-CM may have certain increased effects on the growth of breast cancer *in vitro*.

A scratch wound assay and a transwell assay were conducted to investigate MCF-7 migration. From the statistical data, we hypothesized that 10 and 20% CM may significantly promote MCF-7 cancer cell migration. Our results are consistent with the study by Zhu *et al*(33), which showed that hMSC-CM enhanced tumor growth is sustainable in serial transplantation, indicating that MSC-secreted factors have profound effects on the ‘reprogramming’ of tumor growth.

However, controversies exist concerning the correlations between MSCs and tumors. We speculated that this may be related to the source of the MSCs, individual variations in the physiological immune status of the donors, differences in culture and experimental methods, the type and site of carcinoma, or a combination of these factors. Therefore, studies are required before MSCs may be widely used in clinical cancer therapy, and the mechanism between MSCs, tumorigenesis and tumor progression requires further research. MSCs may provide a new approach for cancer therapy.
